# The impact of headache disorders: a prospective analysis of headache referrals to outpatient and inpatient neurology and emergency services in an Irish University teaching hospital

**DOI:** 10.1007/s11845-023-03425-3

**Published:** 2023-06-27

**Authors:** Sarah Darcy, Emmet Kelly, Denise Choong, Allan McCarthy, Sean O’Dowd, Petya Bogdanova-Mihaylova, Sinéad M. Murphy

**Affiliations:** 1 Department of Neurology, Tallaght University Hospital, Dublin 24 Tallaght, Ireland; 2 Emergency Department, Tallaght University Hospital, Dublin 24 Tallaght, Ireland; 3 Academic Unit of Neurology, Trinity College Dublin, Dublin, Ireland

**Keywords:** Disease burden, Headache, Headache in the emergency department, Inpatient headache management, Migraine, Neurology services

## Abstract

**Background:**

Headache represents a significant proportion of disability globally in general practice, neurology outpatient settings, and emergency departments. There is scant literature regarding the impact of headache on healthcare services in Ireland.

**Aims:**

We aimed to investigate headache burden across the emergency department, inpatient stays, and neurology outpatient department referrals in an Irish University teaching hospital.

**Methods:**

We prospectively collected data regarding emergency department presentations, inpatient neurology consultations, and neurology outpatient referrals for patients with headache between 13th January and 8th March 2020. Data were analyzed using descriptive statistics.

**Results:**

There were 180 emergency department attendances, 50 inpatient consultations, and 76 outpatient referrals with headache. Neurological examinations were often incomplete; neuroimaging was commonly employed. Migraine was the most frequent headache diagnosis at discharge in the emergency department and among inpatients after neurology review. Diagnostic uncertainty was identified—33% of patients left the emergency department with no diagnosis, and “unknown/unspecified headache” was recorded on 49% of outpatient referrals and 30% of inpatient consult requests. Medication overuse headache coexisted with migraine in nine patients in the inpatient group. Prophylaxis had been trialed in 56% of patients with migraine referred to outpatients.

**Conclusions:**

Primary headache disorders have a large impact on hospital services. Diagnostic uncertainty is common; neuroimaging is relied upon. Appropriate care pathways, education, and resource allocation should be prioritized.

**Supplementary Information:**

The online version contains supplementary material available at 10.1007/s11845-023-03425-3.

## Background

Headache disorders carry a lifetime prevalence of around 90% [1], with the most common causes being migraine, tension type headache (TTH), and medication overuse headache (MOH) [2]. Migraine alone is responsible for a large global burden of disability, consistently ranking in the top ten causes of years lived with disability (YLDs) across geographical and socioeconomic borders, with a preferential impact on working-age women [3]. Each million of the European population loses around 400,000 days of work or school per year due to migraine [4]. Headaches account for 2% of emergency department (ED) attendances, with hospital admission frequently occurring due to diagnostic uncertainty or uncontrolled pain [5–7]. Patients often undergo unnecessary neuroimaging and spend many unproductive hours in an environment unsuited to headache care. While only around 2–5% of ED headache presentations are due to a serious underlying cause [8, 9], the fear of missing such a diagnosis weighs heavily, contributing to over-investigation and “just-in-case” admissions.

International guidelines are available to aid the emergency or general medicine practitioner in managing different types of headache disorders. The International Classification of Headache Disorders (ICHD) [10] provides an exhaustive list of diagnostic criteria for the various headache types. Although specific pathways and protocols for the primary, secondary, and tertiary care approach to headache disorders are available [1, 11–14], there is no national consensus guidance for the management of headache patients presenting to secondary and tertiary care in Ireland, a country with a ratio of neurologists to population of 0.76/100,000—the lowest in the developed world and far below a median of 4.84/100,000 across Europe [15]. Neurology outpatient clinics in Ireland are oversubscribed with referrals for patients with headache disorders that could be managed in primary care, which in turn may result in long waiting times [16] that impact upon service provision and increase non-attendance rates [17]. There is little published information available about the burden of headache on hospital services in Ireland.

## Aims and objectives

The objective of this study was to assess the burden of headache disorders on hospital services in Ireland and gain an understanding of current approaches to investigation and management in this setting, to compare against current international recommendations for best practice and to address areas of practice in need of improvement. We aimed to prospectively collect information pertaining to ED attendances with headache, inpatient headache referrals to the liaison neurology service, and referrals to neurology outpatient (OPD) clinic over an 8-week period in an Irish University teaching hospital.

## Methods

In this prospective study, we collected data on all ED presentations with headache and headache-related inpatient consultations (IC) to the neurology service over an 8-week period (13th January–8th March 2020) in Tallaght University Hospital. Of note, the first case of COVID-19 was not identified in the Republic of Ireland until the 29th of February 2020. Subsequent pandemic-related changes to routine hospital functions were not implemented during the study period and are therefore not thought to be relevant. Patient demographic data, referral source, the presence of “red flags” (see supplemental materials, Fig. S1), clinical assessment details, a history of previous ED or neurology OPD attendances, investigations performed, medications administered, discharge diagnoses (primary vs secondary headache), and length of stay (LOS) were recorded for both groups. In the IC group, time from admission to consult request and to subsequent neurology review was also recorded.

Data were obtained for the ED group from electronic records, supplemented with physical chart review where records were either incomplete or missing. Records were examined for information relating to initial assessment by ED doctors, as well as ED-based assessment and initial treatment by the admitting medical (non-neurology) doctors. Relevant records were accessed on a weekly basis through the computer-based ED system (Symphony™) and identified through nursing triage categories allocated to patients for each period. Potentially broadly inclusive triage terms such as “facial pain” and “eye pain” are often used as descriptors for patients presenting with headache to the ED and were therefore included in our triage category list. Clear cases of dental or ear, nose, and throat (ENT) disease, ophthalmological disorders, and trauma were excluded. In the IC group, data were collected from clinical charts, with patients identified daily by the neurology consult team. Results of investigations were accessed via electronic hospital systems for both groups. All referrals to the neurology outpatient clinic over the same time period were reviewed and data recorded from the details provided. The data were analyzed using descriptive statistics. This study was registered with the Clinical Audit Department of Tallaght University Hospital.

## Results

### Emergency department presentations

Seven thousand seven hundred fifty-seven patients were seen in the adult ED during the study period. Two hundred sixty-five of these were initially triaged with “headache,” “facial problem,” or “eye problem.” Eighty-five were excluded from the analysis: 42 had a complaint that was incongruous with the initial triage category or one of an obvious dental, ophthalmological, traumatic or ENT cause; 23 did not wait for review; 11 bypassed ED as direct admission to medical teams, and 9 patients had almost entirely incomplete ED records. The remaining 180 patients (Table 1) represented 2.3% of all ED attendances for the period, including 6 with partially incomplete records, but with largely available data.

**Table 1:** Description of patient demographics and characteristics for all ED attendances

**Characteristic**	***N*** **(180)**	**%**
*Gender*		
Male	60	33.3
Female	120	66.7
*Age–median/range in years*	41/16–85	
16–17	7	3.9
18–49	115	63.9
> 50	58	32.2
*Referral source*		
Self	96	53.3
GP	73	40.6
Ambulance	3	1.7
Other hospital	2	1.1
Unavailable*	6	3.3
*Known primary headache diagnosis*	31	17.2
Previous ED attendance**		
None	135	75
1–3	36	20
4–10	4	2.2
> 10	1	0.6
Unavailable*	4	2.2

^*^Partially incomplete records

^a^With headaches only

Sixty seven patients (37%) had at least one “red flag.” Of these, 61 (91%) had a CT brain, 56 (84%) were admitted, and 14 (21%) underwent a lumbar puncture. Eleven (17.8%) of those with “red flags” who had imaging had a secondary cause identified. In the entire ED cohort, 57% (*n* = 103) had a CT brain and a secondary cause was found on imaging in just 12 patients overall, or in 11.7% of all patients who underwent a CT brain.

Most patients who were seen in ED and subsequently discharged (*n* = 89/ 49.4%) underwent an examination of limbs (tone, power, coordination, sensation, and deep tendon reflexes), cranial nerves and gait, but the vast majority had no documented examination for meningism, and fundoscopy was only attempted in one patient (Table 2). The most commonly administered medications in the ED setting were paracetamol, non-steroidal anti-inflammatory drugs (NSAIDs), and anti-emetics. Patients seen and discharged in ED who underwent neuroimaging (CT brain in 19/89 patients, 21.3%) had no secondary causes for headache identified. Of fifteen patients, > 50 years with new headache, 2 (13%), had an ESR checked prior to discharge. It is not routine in our hospital for patients to be reviewed by neurology in the ED prior to admission, although there may be some exceptions to this practice at times. However, there was no documentation of neurology input prior to discharge for patients seen in the ED who were not admitted during this study period.

**Table 2:** Details of initial assessment/management by ED staff and non-neurology medical teams for patients discharged from ED^a^

	***N* (89)**	**%**
*Examination*		
Documentation of at least partial neurological exam^b^	73	82
Fundoscopy	1	1.1
Documentation for meningism	22	24.7
*Medications administered*		
Paracetamol	44	49.4
NSAID	32	36
Anti-emetic	27	30.3
No medication	28	31.5
Opiate	10	11.2
Triptan	3	3.4
Other^c^	2	2.2
Oxygen	1	1.1
Data unavailable	1	1.1
*Neuroimaging*		
CT brain	19	21.3
No imaging	70	78.7

^a^Discharge destination home (*n* = 88) or other hospital for investigation (*n* = 1)

^b^Comprising assessment of any/all of the following: cranial nerves, tone, power, coordination, sensation, reflexes, and gait

^c^Benzodiazepine (*n* = 1) and steroid (*n* = 1)

**Fig. 1:**
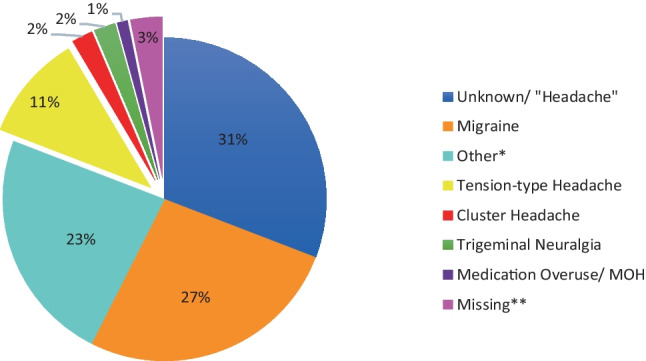
Final diagnosis made in ED prior to discharge. *Other diagnoses recorded comprised upper respiratory tract infections, hypertension, post-concussion syndrome, musculoskeletal disorders, otolaryngological causes, anxiety, “pregnancy,” and ventriculoperitoneal shunt dysfunction. **3 records could not be located

Migraine was both the most common initial and final diagnosis given in ED prior to discharge (Fig. 1). Diagnoses were often unknown, not documented, or recorded as “headache.” TTH was the diagnosis at discharge for ten patients (11.2%) although it is important to recognize that ED diagnoses may not align with expert neurological opinion. There was a single diagnosis of “MOH,” but this patient also had a background history of migraine, which was likely the primary underlying condition driving medication overuse and ED attendance. Ninety one patients (50.5%) were admitted from ED, for whom the median LOS was 3 days (IQR 3.5). Eighty eight patients (48.9%) were discharged home without admission after review by ED staff, and 1 patient was transferred to another hospital for specialist management.

### Inpatient consults

Over the 8-week period of data collection, the neurology service received 119 consultation requests and 50 of these were related to headaches (Table 3). No patients were excluded from data analysis, although one record was partially incomplete. All 17 patients (34%) with a documented “red flag” underwent either CT or MR neuroimaging or both. Most (31/33) of the remaining patients also had neuroimaging during their admissions. Two patients had idiopathic intracranial hypertension, one had giant cell arteritis, and one had encephalitis. In all of these cases, imaging was supportive of clinical diagnoses, not diagnostic in isolation. Although all patients had at least a partial neurological examination performed by referring medical teams *prior* to neurology consultation, the majority did not undergo an examination of gait, meningism, or of the fundi (Table 4). Paracetamol and NSAIDs were the most common analgesic options administered during inpatient stays. 42.9% of patients ≥ 50 years with a new headache presentation had an ESR checked.

**Table 3:** Characteristics of patients referred to neurology consult service within the 8-week audit period

**Characteristic**	***N*** ** (50)**	**%**
*Gender*		
Male	15	30
Female	35	70
*Age–median/range in years*	44/16–74	
16–17	1	2
18–49	32	64
> 50	17	34
*Time from admission to consult request*		
Same day	22	44
1–2 days	23	46
3–5 days	5	10
*Time from consult request to neurology review*		
Same day	30	60
1 day^a^	18	36
2–3 days	2	4
*Known primary headache diagnosis*	9	18
*Previously known to neurology service*	7	14

^a^Requests updated at 8 am and 1 pm

**Table 4:** Workup completed by inpatient medical teams prior to referring patients for neurology consultation

	***N***	**%**
*Examination (n = 49*^a^*)*		
Documentation of at least a partial neurological examination^b^	49	100
Fundoscopy	7	14.3
Documentation for meningism	16	32.7
*Medications administered (n = 49)*		
Paracetamol	41	83.7
NSAID	20	40.8
Anti-emetic	8	16.3
Opiate	7	14.3
Triptan	6	12.2
No medication	5	10.2
*Neuroimaging (n = 50)*		
CT Brain	47	94
MRI Brain	22	44
Vascular imaging^b^	15	30
No imaging	2	4
*ESR (> 50 years with new headache, n = 14)*		
Tested	6/14	42.9
Abnormal	4/6	66.7

^a^One record of examination and medications administered could not be located

^b^Comprising assessment of any/all of: cranial nerves, tone, power, coordination, sensation, reflexes, gait

^c^CT angiography (*n* = 7), MR venography (*n* = 5), carotid doppler ultrasound (*n* = 3), CT venography (*n* = 1)

**Fig. 2:**
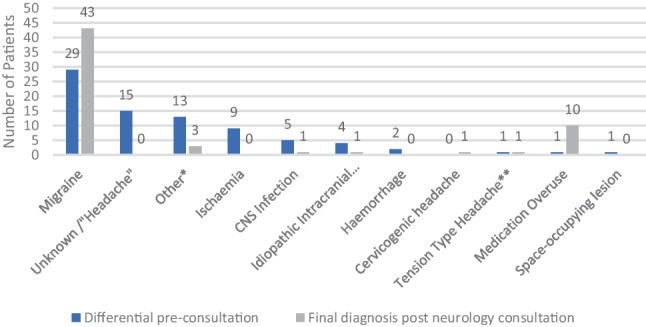
Differential diagnoses pre-consult vs final diagnoses. * “Other” differential diagnoses comprised viral illness, hypertension, multiple sclerosis, seizure, arterial dissection, and giant cell arteritis. **In the context of mental health difficulty

Migraine was the most common differential diagnosis considered by the primary/admitting team (*n* = 29, 58%). Migraine was also by far the most common discharge diagnosis post-neurology consult (*n* = 43, 86%) and was associated with MOH in 21% of these cases (*n* = 9). MOH was identified as a potential differential by the inpatient team prior to neurology review in only one patient (Fig. 2).

### Outpatient referrals

There were 76 outpatient referrals to the headache clinic over the period (Table 5). Referrals were often extremely brief and lacking in important information. Unsurprisingly, there was predominance of referrals for females aged 16–49 years. Referrals were largely (83%) from general practitioners (GPs) with the remainder from other hospitals and other specialties within the same hospital. A minority (33%) documented relevant neurological examination findings. Patients had a median 28-week history of symptoms at the time of referral (IQR = 165 weeks).

**Table 5:** Characteristics of patients referred to the headache clinic over the 8-week study period

	***N* (76)**	**%**
*Gender*		
Male	21	27.6
Female	55	72.4
*Age*		
16–49yrs	52	68.4
≥ 50yrs	24	31.6
*Referral source*		
GP	63	82.9
Other specialty same hospital	6	7.9
Different hospital	7	9.2
*Previously seen by Neurology service*	11	14.5
*Imaging to date (ordered or completed)*		
CT	32	42.1
MRI	31	40.8
Vascular Imaging^a^	6	7.9
None	29	38.2
*Acute medications used prior to referral*		
Paracetamol	24	31.6
NSAID	22	28.9
Triptan	13	17.1
Opioid	9	11.8
None	33	43.4
*Number of prophylactic trials*^*b*^		
One	18	23.7
Two	3	3.9
Three	1	1.3
Four	1	1.3
None	55	72.4

Information regarding length of trial/ dose was not available

^a^2 MR angiograms, 1 Carotid doppler ultrasound, 2 CT brain scans, 1 patient with MR angiogram, MR venogram, and CT angiogram

^b^Prophylactic agents trialed included amitriptyline, propranolol, pregabalin, flunarizine, candesartan, and topiramate

Referrals were reviewed by a neurologist and triaged accordingly. “Red flag” symptoms and signs were initially reported by referrers in 30 cases (39.5%); however, following review of the referral details by a neurologist were deemed to represent true “red flags” in 14 cases (18.4%). The most common referral question was “unspecified headache” (*n* = 37, 48.7%), followed by migraine (*n* = 32, 42.1%). The remainder consisted of trigeminal neuralgia (*n* = 2), trigeminal autonomic cephalalgia (*n* = 2), functional neurological disorder (*n* = 1), “primary” medication overuse headache (*n* = 1), and tension type headache (*n* = 1). Most patients had already had imaging (usually reassuring), yet a minority (28%) had trialed any prophylactic medication despite 42% being referred with migraine.

## Discussion

In this study, we prospectively collected data on all patients presenting with headache to ED, all inpatient headache referrals to the neurology consult service, and all headache referrals to the neurology OPD over an 8-week period. We aimed to describe the approach to the patient with headache in non-specialist settings (ED and GP) in terms of initial examination, differential diagnosis, investigations, and initial treatments. We also aimed to identify the underlying disorders driving headache presentations to the ED, hospital admissions, and referrals for neurology consultation and OPD assessment. There are little data regarding the impact of headache on the Irish health service, but it is the most common neurological complaint raised at GP visits [18], one of the most frequent reasons for referrals to neurology clinics [16, 19], represents around 2% of all ED visits [9], and is estimated to cost the Irish exchequer €290 million per annum [15]. We aimed to illustrate the burden of headache across neurology and emergency services in an Irish University teaching hospital.

Our data demonstrate a clear preponderance of migraine as a cause of clinically significant headaches, and a majority working-age female population seeking emergency and specialist care—a trend that has been borne out by global figures compiled to date [3, 20]. Headache presentations made up a large proportion—42%—of all neurology consults received over the study period when compared with previous studies of neurology consult services in Ireland that reported this figure between 12 and 16.5% [21].

The uncertainty that surrounds the diagnostic process for headache was reflected by differentials often recorded as “unknown” or “unspecified headache.” Thirty-three percent of patients discharged by the ED team were given a diagnosis of “headache” or “unknown.” This phenomenon of diagnostic uncertainty has been observed on a wider scale across all neurological presentations to the ED but is particularly prevalent in the diagnosis of primary headache disorders [22]. “Unspecified headache” was also the most offered presumptive diagnosis among outpatient referrals. The ICHD-3 “headache unspecified” classification (14.2) that this category aligns with most closely should only be used “where information cannot be obtained because the patient is dead, unable to communicate, or unavailable [10].” Awareness of the common exacerbating issue of MOH that is frequently associated with migraine [23] is poor, as it formed part of the initial differential in no ED attendances and in only one inpatient prior to neurology review. However, findings from our study imply that it may be an under recognized driver of disabling migraine and subsequent hospital attendance and admission.

Examinations completed by ED and inpatient medical teams were often lacking in essential components. Although most patients in both the ED and IC groups had an at least partial neurological examination documented, specific elements were frequently overlooked. Examinations of gait and for meningism were often omitted. Perhaps, most striking however is the lack of attempts at fundoscopy which was carried out for only one patient in ED (where ophthalmoscopes are readily available) and for just seven inpatients prior to neurology consult request. The British Association for the Study of Headache (BASH) guidance [14] indicates that fundoscopy to evaluate for papilloedema is an indispensable part of the examination of the patient presenting with headache, but there is a growing reluctance to perform it and a lack of confidence in its interpretation [24]. Admission proformas in our ED also have a specific section for fundoscopy yet it was still not attempted in the majority. Only a third (32.9%) of patients referred to the outpatient clinic had a neurological examination of any kind documented in their referral. There are diverse potential reasons for the lack of a complete neurological examination in these settings, including time constraints—for example, the average length of GP consultation in Ireland is around 14 min [25]. “Neurophobia” may also be a factor—the term coined for the perception (initially among medical students) that clinical neurology and neuroscience are complex, poorly taught, and difficult to integrate [26]. It has been demonstrated that this view does not end with medical school and is observed among hospital and GP trainees [27, 28]. This is a larger issue with implications for future training and recruitment in neurology that needs to be addressed in the early stages of medical education.

The use of opiate medications in the management of headaches has been identified as a major problem internationally [7]. First-line use of opioids for migraine, the most common headache disorder encountered in ED and inpatient consultations, is associated with longer hospital stays, inadequate symptom relief, and the use of further additional medications to control symptoms [29]. It was therefore reassuring to see opiates prescribed in a minority of cases in ED (11%), for inpatients (14%), and among outpatient referrals (11.8%). However, our group has previously found that non-prescribed opiate use is frequent in patients attending the neurology clinic with headache [16]. Avoidance of opiate medications in this population must remain a strong message to all health practitioners involved in the management of migraine and headache disorders to reduce these numbers even further. Instigation of prophylactic medications where indicated may be helpful here. It is worth noting that although it is recommended that prophylaxis be used where acute medications are ineffective/contraindicated or are overused [1], appropriate preventative agents had been trialed in just under 30% of patients referred to clinic in this study.

Most ED attendees and inpatients underwent neuroimaging, mainly consisting of a CT brain in the first instance. CT is most useful in the acute investigation of headaches with presumed emergent underlying aetiologies; the American Headache Society recommends that MRI should otherwise be the first imaging modality of choice where imaging is required [12]. It is beyond the scope of this study to comment on the appropriateness of neuroimaging for individual patients; however, it is worth noting that 94% of patients in the IC group had a CT brain booked by the referring team, either prior to MRI or as the only imaging technique. The diagnostic yield of imaging stood at 4% in this group. This is higher than the yield observed in patients seen in a specialty outpatient headache service (2.1%) [30] but is in line with that reported in patients presenting to the ED in other studies [31, 32]. Twenty one percent of patients who were ultimately discharged by the ED team had a CT brain, with no serious secondary causes identified. The diagnostic yield from neuroimaging among the entire ED cohort was 11.7%, with etiologies being mainly vascular and these patients usually had other concerning symptoms such as persistent vomiting and focal neurological signs. One recent multinational observational study [23] of non-traumatic headache presentations to the ED found that overall, CT scans were performed in 36.6% of patients with an etiology identified in 9.9% of these. There was large variation between countries; however, our findings are in line with the UK (44.6% undergoing CT, 11.4% clinically significant abnormality found). Serious secondary causes mostly comprised subarachnoid hemorrhage, other hemorrhage, stroke, neoplasm, and meningitis which are broadly in keeping with our findings [23]. Where imaging is desirable, in the ED setting, CT is more readily available than MRI, but this should not automatically justify the use of CT as a substitute when it is unlikely to yield relevant clinical information and/or an MRI is likely to be necessary. There are issues with a blanket CT-based approach that include unnecessary cost, wait times, radiation dose, non-clinically significant findings, as well as a false sense of reassurance due to the inadequacy of CT imaging for areas such as the pituitary or posterior fossa. The diagnosis of headache disorders should primarily be driven by a careful history and examination wherever possible—not by a “negative” or inappropriate CT scan [13]. Evidence suggests that a careful reduction of the number of CT brain scans performed for headache in the ED setting is not associated with increased missed diagnoses or death [33].

Patients referred to the headache clinic had a varying duration of symptoms prior to referral, ranging from 1 week to 22 years. The average waiting time for an outpatient appointment at the general neurology clinic in this hospital at the time of the study was 3 years [16]. It is crucial then, that GPs and patients have the necessary skills, tools, and resources to manage primary headache disorders effectively in the community. Keeping patients with chronic primary headache disorders out of EDs and hospitals should be a priority that is grounded in a desire for better patient outcomes, health systems efficiency, and good economic sense. The vast majority of people with common headache disorders are best managed in primary care settings [34]. One way to reduce ED and hospital attendances is to move the focus towards GP care with access to outpatient headache clinics and imaging where necessary. It is important to note however that there may be significant financial barriers to GP-led care for Irish patients who do not qualify for free appointments under the General Medical Services (GMS) scheme. It is free to attend specialist hospital clinics in the public system following a GP referral in Ireland, which may influence patient preference. In the UK, where GP visits are free, one study found that 85% of GPs surveyed would manage chronic migraine without referral to a specialist, and that 94% would prescribe preventative treatment when needed [35]. Increasing waiting times for GP appointments in Ireland may also contribute to a larger number of patients attending ED. Routine GP appointments can currently have a wait time of up to 2 weeks [36]. There is evidence that community-based care can be effective within 6 months when combined with patient education, physician training, and adequate follow-up [37]. As a step in this direction, our hospital has put together a detailed headache referral pathway including diagnostic criteria and first-line management options that is now available online to GPs and patients [38]. A similar pathway for emergency and inpatient headache management, combined with training and education sessions, would also be helpful to improve ED and inpatient headache assessment and management going forward.

Migraine was by far the most common diagnosis driving ED attendances, inpatient consultations, and outpatient referrals in this study. This is unsurprising considering its status as a leading cause of disability-adjusted life years and years of healthy life lost due to disability in Europe and globally. Despite this, people with migraine remain an underserved population when compared to those with other common neurological disorders like epilepsy, for which there has been an overall decrease in disease burden [3], attributed to improved access to treatment over the last 2 decades. There has been no such change in migraine-related disease burden [39]. Access to specialist headache care in Ireland has significant barriers with often extraordinarily long waiting lists due to limited resources and high demand. Integrated care pathways for epilepsy care in the ED can result in improved clinical evaluation and documentation, as well as reduced LOS and readmission rates [40]—the application of similar pathways for headache presentations in tertiary centers should be strongly considered and ideally implemented in a standardized way across all Irish hospitals to ensure continuity of care and compliance from medical staff who move between centers frequently throughout clinical training. Resources have recently been allocated through the Sláintecare Integration Fund in Ireland to develop and implement a community-based program to improve headache management by GPs and to empower and inform patients towards self-management [41]. It is hoped that this will reduce the current pressure on secondary and tertiary care. The Irish College of General Practitioners has also launched a reference guide for the management of migraine in primary care, which was available to GPs at the time of our data collection [42]. Our study indicates that despite these steps, there is still a need for more community-based support for headache diagnosis and management. This study has limitations. Seasonal bias cannot be out ruled, and further data collection was constrained for pandemic reasons. It was often outside the study scope to comment on clinical reasoning in individual cases which limits the interpretation of some data.

## Conclusion

This study has documented the burden of headache disorders on the hospital service in Ireland. Diagnostic uncertainty is common and likely contributes to over-investigation and defensive medicine, as well as frequent and perhaps unnecessary outpatient referrals to clinics that are already oversubscribed. Incomplete neurological examinations were observed across all groups and may be reflective of time pressures or neurophobia, while frequent and low yield use of neuroimaging may reflect a defensive or overly cautious practice that is not without potential harm. A positive finding was the comparatively low rates of opiate use as first-line treatment options when compared with the USA; however, more work can still be done in this area. Although specialist clinics are currently operating at unsustainable levels, there has been a national commitment to improving and integrating the primary, secondary, and tertiary models of headache care. This must include clear dedicated care pathways, appropriate resource allocation, and a multifaceted education and training approach that involve patients, primary care practitioners, nurses and nurse specialists, ED, and hospital physicians in order to streamline and improve headache care in Ireland.

### Supplementary Information

Below is the link to the electronic supplementary material.

Supplementary file1 (DOCX 15 KB)

## Data Availability

The data that support the findings of this study are not openly available due to reasons of sensitivity and are available from the corresponding author upon reasonable request. Data are located in controlled access data storage at Tallaght University Hospital.
