# Supplementary material for: The impact of headache disorders: a prospective analysis of headache referrals to outpatient and inpatient neurology and emergency services in an Irish University teaching hospital
Source: Ir J Med Sci. 2023 Jun 27;193(1):397–405. doi: 10.1007/s11845-023-03425-3 (PMC10808417; doi:10.1007/s11845-023-03425-3)
Supplement: Supplementary file 1 — Supplementary file1 (DOCX 15 KB) [file 11845_2023_3425_MOESM1_ESM.docx]

**Figure S1**

| List of Qualifying Red Flags^1^ |
| --- |
| - Thunderclap headache - Papilloedema - Focal Neurological signs - Evidence of raised intracranial pressure ^2^ - Seizures - Fever - Meningism - Reduced alertness (Glasgow Coma Scale) - Red eye/visual loss - New headache >50 years +/- raised erythrocyte sedimentation rate (ESR) - New Headache >8 weeks or progressively symptomatic - Pregnant >20 weeks or early postpartum - Cognitive or behavioral changes - Head trauma in preceding three months - Malignant range (>180/120mmHg) hypertension - History of malignancy - History of immunosuppression e.g., immunosuppressive drugs, HIV |

*^1^ Adapted from:* *Headaches in over 12s: diagnosis and management. London: National Institute for Health and Care Excellence (NICE); 2021 Dec 17*

*^2^ Including headache worsened by Valsalva or waking from sleep, vomiting, pulse synchronous tinnitus and postural visual loss.*
